# Moderation of Calpain Activity Promotes Neovascular Integration and Lumen Formation during VEGF-Induced Pathological Angiogenesis

**DOI:** 10.1371/journal.pone.0013612

**Published:** 2010-10-25

**Authors:** Mien V. Hoang, Janice A. Nagy, Joan E. B. Fox, Donald R. Senger

**Affiliations:** 1 Department of Pathology and Center for Vascular Biology Research, Beth Israel Deaconess Medical Center and Harvard Medical School, Boston, Massachusetts, United States of America; 2 Department of Molecular Cardiology, Lerner Research Institute, Cleveland Clinic, Cleveland, Ohio, United States of America; University of Giessen Lung Center, Germany

## Abstract

**Background:**

Successful neovascularization requires that sprouting endothelial cells (ECs) integrate to form new vascular networks. However, architecturally defective, poorly integrated vessels with blind ends are typical of pathological angiogenesis induced by vascular endothelial growth factor-A (VEGF), thereby limiting the utility of VEGF for therapeutic angiogenesis and aggravating ischemia-related pathologies. Here we investigated the possibility that over-exuberant calpain activity is responsible for aberrant VEGF neovessel architecture and integration. Calpains are a family of intracellular calcium-dependent, non-lysosomal cysteine proteases that regulate cellular functions through proteolysis of numerous substrates.

**Methodology/Principal Findings:**

In a mouse skin model of VEGF-driven angiogenesis, retroviral transduction with dominant-negative (DN) calpain-I promoted neovessel integration and lumen formation, reduced blind ends, and improved vascular perfusion. Moderate doses of calpain inhibitor-I improved VEGF-driven angiogenesis similarly to DN calpain-I. Conversely, retroviral transduction with wild-type (WT) calpain-I abolished neovessel integration and lumen formation. *In vitro*, moderate suppression of calpain activity with DN calpain-I or calpain inhibitor-I increased the microtubule-stabilizing protein tau in endothelial cells (ECs), increased the average length of microtubules, increased actin cable length, and increased the interconnectivity of vascular cords. Conversely, WT calpain-I diminished tau, collapsed microtubules, disrupted actin cables, and inhibited integration of cord networks. Consistent with the critical importance of microtubules for vascular network integration, the microtubule-stabilizing agent taxol supported vascular cord integration whereas microtubule dissolution with nocodazole collapsed cord networks.

**Conclusions/Significance:**

These findings implicate VEGF-induction of calpain activity and impairment of cytoskeletal dynamics in the failure of VEGF-induced neovessels to form and integrate properly. Accordingly, calpain represents an important target for rectifying key vascular defects associated with pathological angiogenesis and for improving therapeutic angiogenesis with VEGF.

## Introduction

Vascular endothelial growth factor-A (VEGF) is essential for embryonic vasculogenesis and for angiogenesis in a variety of important pathologies including ischemia and wound repair, proliferative retinopathies, psoriasis, rheumatoid arthritis, and cancers [1]. Paradoxically, VEGF induces a highly abnormal vasculature in pathological settings. Typical vascular abnormalities include vessel tortuosity, abnormal vessel spacing and branching, vessel leakiness, and failed integration of vascular networks resulting in numerous blind ends (reviewed in [2], [3]). These architectural defects result in poor blood vessel function, especially poor blood flow. Vascular abnormalities may be due to comparatively high VEGF expression in pathological settings [4] and also may result from an imbalance between VEGF and other important factors [2], including the vascular cytokine angiopoietin-1 [5]. Regardless, cellular mechanisms responsible for abnormal neovascularization in pathological settings have been largely unexplored.

In searching for mechanistic explanations for the architectural defects associated with abnormal angiogenesis, we became interested in a possible connection between failed integration of neovascular networks, VEGF, and calpain activity. Calpains are intracellular, calcium-dependent thiol proteases (reviewed in [6]). Upon activation, these widely expressed enzymes cleave a broad spectrum of functionally important intracellular protein targets [6] that regulate cytoskeletal organization [7], cell adhesion and spreading [8], [9], [10], and cell migration [10], [11], [12]. Moreover, VEGF has been shown previously to induce calpain activity in endothelial cells (ECs) [13], [14]. Therefore, we directly examined the involvement of calpain activity in regulating the integration of VEGF-induced neovascular networks *in vivo* with both genetic and pharmacologic approaches. We also employed *in vitro* models of capillary morphogenesis to explore, at the cellular level, mechanisms by which calpain activity controls the assembly and organization of ECs into new blood vessels. Our findings directly implicate VEGF-induction of calpain activity in the failed inter-connectivity of neovascular networks and illustrate that appropriate inhibition of calpain substantially improves neovessel integration and lumen formation.

## Results

### A Dominant-negative Mutant of Calpain-I Improves VEGF Neovessel Integration and Lumen Formation *In Vivo*

To investigate a possible relationship between calpain activity and abnormal neovascular architecture, we employed an established mouse model of VEGF-driven angiogenesis [15], [16]. This model utilizes immortalized, transfected cells engineered for continuous expression and secretion of VEGF_165_ under the direction of a constitutively active cytomegalovirus immediate-early gene (CMV) promoter. To provoke angiogenesis, the VEGF-expressing cells are mixed with basement membrane Matrigel, and the mixture is injected into the sub-dermal space. Robust angiogenesis in the overlying dermis is typically evident by four days, and the neovasculature routinely exhibits all of the hallmarks of pathological angiogenesis (e.g., vessel tortuosity, abnormal vessel spacing and branching, vessel leakiness, and failed integration of vascular networks resulting in numerous blind ends). In addition to the VEGF-expressing cells, we included equal numbers of retroviral packaging cells in the Matrigel for continuous delivery of engineered retroviruses. The different retroviral packaging cells expressed retrovirus encoding either a validated dominant-negative (DN) mutant of calpain-I [8], wild type (WT) calpain-I [8], or no insert (empty vector). This retrovirus-based model of VEGF-driven angiogenesis offers the advantage of high retroviral transduction efficiency that is favored in proliferating cells, and ECs are actively dividing in response to continuous VEGF-stimulation [15], [16]. Moreover, the inclusion of packaging cells provides a constant source of freshly produced retrovirus throughout the experimental interval. Previously, the efficacy of this model has been validated with packaging cells expressing retrovirus encoding GFP [16], RhoA mutants [15], and transcription factor Nur77 [17].

**Figure 1:**
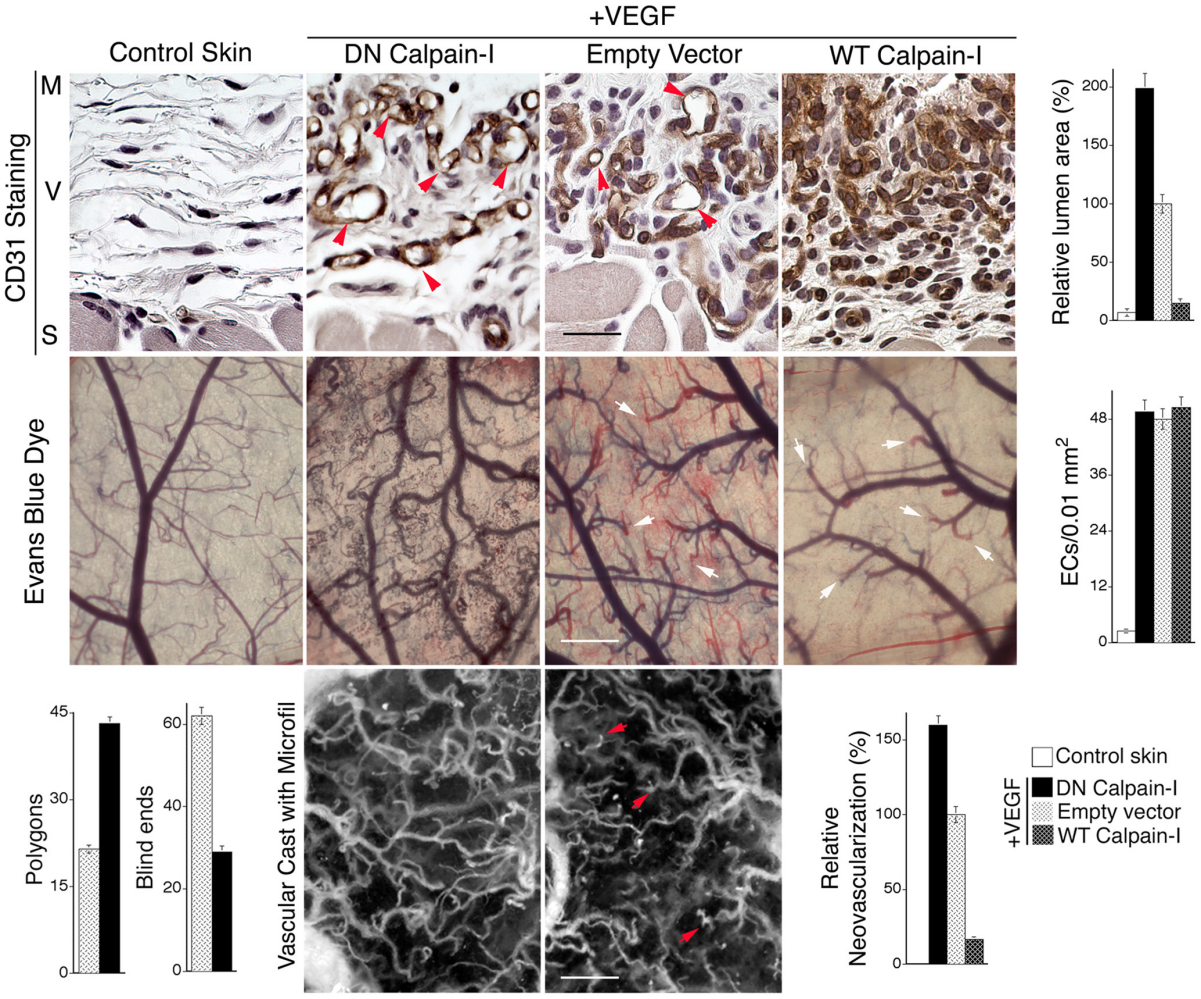
DN calpain-I improves VEGF neovessel lumen formation and integration *in vivo*. Transfected SK-MEL2 cells engineered to express VEGF_165_ were mixed with retroviral packaging cells expressing retroviruses encoding either dominant-negative (DN) calpain-I, wild-type (WT) calpain-I or control retrovirus (Empty Vector), as indicated, and injected together with Matrigel into the sub-dermal space as described in Materials and Methods. After 8 days, the structure and function of the angiogenic response elicited in the overlying dermis was investigated. Animals were harvested following 10 min perfusion with the indicated tracer and tissue sections were analyzed by immunohistochemistry. CD31 Stain: Staining of ECs in cross section with CD31 antibody (brown color) illustrates that DN calpain-I improved lumen formation (red arrowheads) relative to Empty Vector control, whereas WT calpain-I almost completely abolished lumen formation. Scale bar  = 25 microns. M  =  Matrigel, V  =  region of neovascularization, S  =  skeletal muscle. Evans blue dye: Gross images of the vasculature in the dermis overlying the Matrigel implants (scale bar  = 500 microns) show that DN calpain-I improved blood vessel integration and tracer perfusion (Note: all perfused blood vessels appear blue) and reduced blind ends (white arrows) relative to Empty Vector control (Note: numerous red-tipped vessels indicating sprouts that have not been perfused with Evans Blue tracer), whereas WT calpain-I blocked formation of new blood vessels. Vascular Cast with Microfil: Following perfusion of the entire vasculature with Microfil, blind ends (red arrows) were found to be numerous in the Empty Vector control but vessel inter-connectivity was substantially improved by transduction with DN calpain-I. Scale bar  = 450 microns. Bar graphs present quantification of relative lumen area from CD31-stained sections; n≥18 for each group, p<0.01 for DN calpain-I vs. empty vector and WT calpain-I vs. empty vector; ECs (per 0.01 mm^2^) from CD31-stained sections; n≥18 for each group; relative neovascularization (from gross images); n≥18 for each group; p<0.01 for DN calpain-I vs. empty vector and WT calpain-I vs. empty vector; and quantification of polygons (closed vascular loops) (p<0.02) and blind ends (p<0.02) from vascular casts with Microfil; ≥10 for each group. Values reported for polygons and blind ends correspond to a sample area of 12 mm^2^.

Animals were harvested on day 8 following induction of angiogenesis, at which time neovascularization of the over-lying dermis was extensive. As shown with CD31-staining of paraffin sections (Fig. 1, top panels), ECs in the VEGF + DN calpain-I specimens were well organized into blood vessels with clearly distinguished lumens, whereas ECs in the VEGF + WT calpain-I group were very poorly organized and lumens were nearly absent. ECs in VEGF + empty vector specimens exhibited intermediate lumen formation (Fig. 1, top panels, Fig. 1 bar graph: Relative lumen area; for lower power views of larger fields see Fig. S1, Supporting Information). Interestingly, the numbers of ECs per unit area in cross-section was indistinguishable among DN calpain-I, WT calpain-I, and empty-vector groups (Fig. 1 bar graph: “EC density”), indicating that expression of DN calpain-I or WT calpain-I had no influence on EC number. Consistent with these observations, co-culture of the various retroviral packaging cells with the VEGF-expressing SK-MEL-2 cells in the same proportions employed *in vivo* had no effect on VEGF production (see Methods), as expected because VEGF expression in this system is constitutively driven by a CMV promoter. Thus, the marked differences in lumen formation among the different experimental groups can best be explained by differences in blood vessel formation rather than differences in EC density or VEGF expression.

Consistent with the CD31-staining analyses, microscopic analyses of the vasculature in whole mounts revealed that prominent neovessel sprouts in the VEGF + DN calpain-I group were well integrated and perfused by Evans blue dye, whereas neovessel sprouts in the VEGF + empty vector group were poorly integrated, exhibited blind ends, and were incompletely perfused (Fig. 1 Evans Blue Dye). In sharp contrast, neovessels in the VEGF + WT calpain-I group were virtually absent indicating total disruption of angiogenesis by WT calpain-I. Quantification of perfused neovessel density from gross images taken at dissection indicated that DN calpain-I increased neovascularization 60% relative to empty-vector controls; whereas, WT calpain-I inhibited neovascularization by >80% (Fig. 1 bar graph: “Relative neovascularization”). Perfusion with lysine-fixable 70 kD Texas-red dextran and confocal microscopy confirmed the architectural distinctions observed with Evans blue perfusion (Supporting Information, Fig. S1).

To analyze further the improvement in angiogenesis attributable to DN calpain-I, we prepared vascular casts with Microfil perfusion (Fig. 1, Vascular Cast with Microfil). Two parameters were quantified: numbers of neovessels with blind ends and neovessel integration with neighboring neovessels as measured by counting closed vascular loops (polygons) [18]. These quantitative analyses indicated that, relative to empty-vector, DN calpain-I reduced blind ends >50% and increased the number of closed vascular polygons >100%, thus indicating substantial improvement in vascular integration (Fig. 1 bar graphs: “Polygons” and “Blind ends”).

### DN Calpain-I Supports Integration of Vascular Networks During Capillary Morphogenesis *In Vitro*

**Figure 2:**
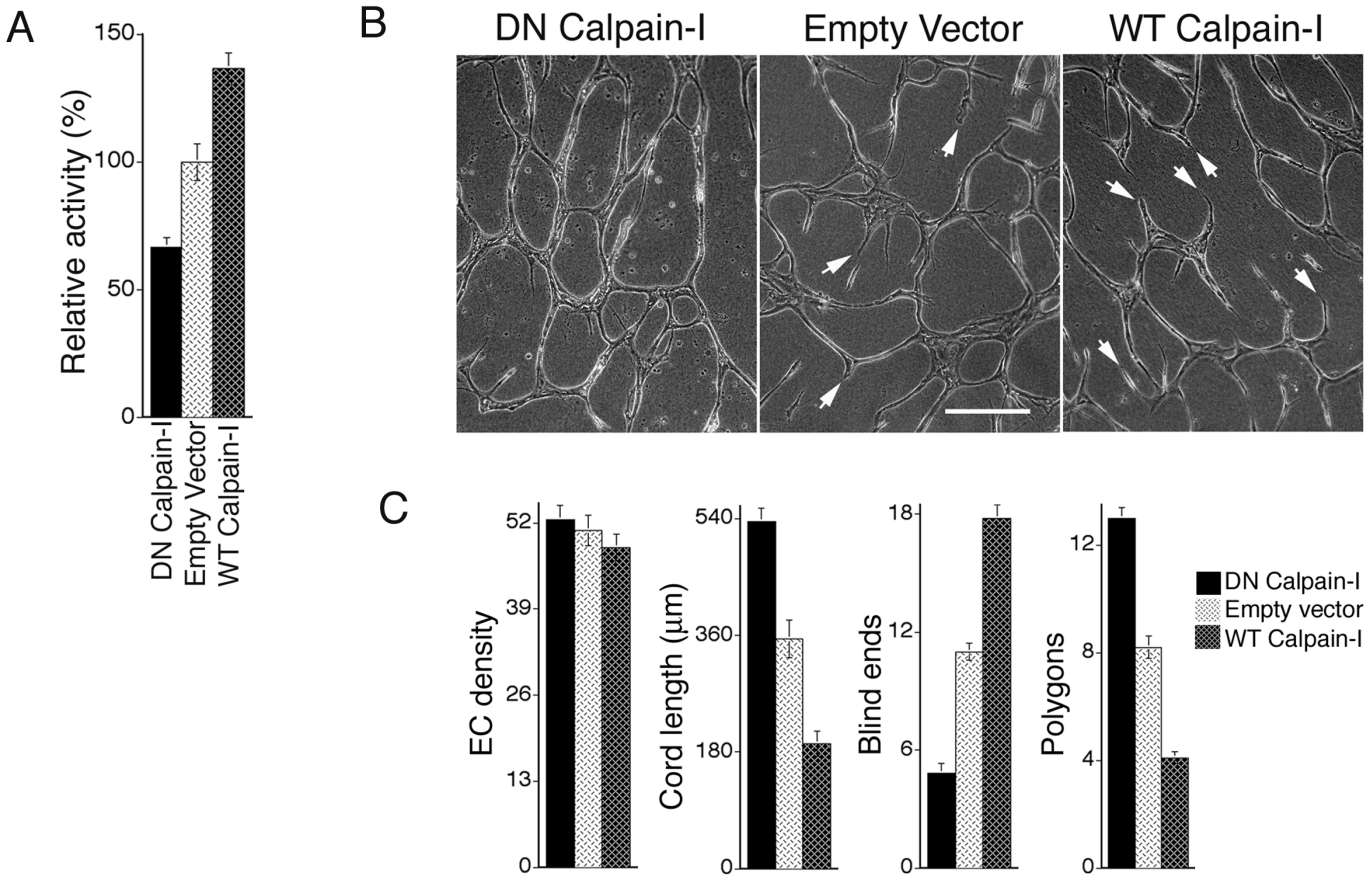
Calpain activity regulates integration of vascular networks during capillary morphogenesis *in vitro*. (A) Quantification of relative calpain activity, as determined with a fluorescent calpain substrate assay (see Methods) in MVECs transduced with DN calpain-I, empty vector, or WT calpain-1 (n>17 and p≤0.01 for all comparisons). (B) Equal numbers of transduced dermal MVECs (+20 ng/ml VEGF) were overlaid with three-dimensional collagen-I to induce assembly of vascular cords. Relative to empty vector control, transduction with DN calpain-I enhanced inter-connectivity of vascular cords. In contrast, WT calpain-I increased blind ends (white arrows) and diminished cord inter-connectivity. Bar  = 100 µm. (B) Quantification of EC density and cord organization; n≥17 for each group; measured parameters correspond to actual areas of 0.4 mm^2^. Cord length: p<0.001 for DN calpain-I vs. empty vector and WT calpain-I vs. empty vector. Blind ends: p<0.002 for DN calpain-I vs. empty vector and WT calpain-I vs. empty vector. Polygons (closed vascular loops): p<0.001 for DN calpain-I vs. empty vector and WT calpain-I vs. empty vector.

To investigate cellular mechanisms through which calpain controls neovessel formation and integration, we prepared cultured human dermal microvascular ECs (MVECs) transduced with DN calpain-I, WT calpain-I, and empty vector. Cells were cultured in the continuous presence of VEGF to imitate conditions *in vivo*. As expected, MVECs transduced with DN calpain-I exhibited a moderate but significant decrease (∼35%) in calpain activity, relative to controls, as measured with a fluorescent calpain substrate assay (Fig. 2A, see Methods). Conversely, MVECs transduced with WT calpain-I exhibited a moderate but significant increase in activity (Fig. 2A). Next, these MVEC populations were stimulated with three-dimensional collagen-I matrices, thereby inducing assembly of vascular cord-like structures that are the precursors to tubes with lumens [19], [20]. This dynamic process closely resembles formation of blood vessels *in vivo* [21], [22]. Consistent with the *in vivo* experiments described above, transduction of dermal MVECs with retrovirus carrying DN calpain-I enhanced the inter-connectivity of vascular cords *in vitro* (Fig. 2B, C). DN calpain-I increased cord length, reduced blind ends, and increased formation of closed polygon networks relative to empty-vector controls (Fig. 2B, C). Conversely, transduction with retrovirus carrying WT calpain-I reduced cord length, increased the number of blind ends, and reduced formation of closed polygon networks (Figs. 2B, C). Importantly, these findings established direct parallels with our observations *in vivo* (Fig. 1), thereby confirming the value of this *in vitro* assay for further investigations on mechanism.

### DN Calpain-I Supports Vascular Cord Integration by Improving Cytoskeletal Dynamics

**Figure 3:**
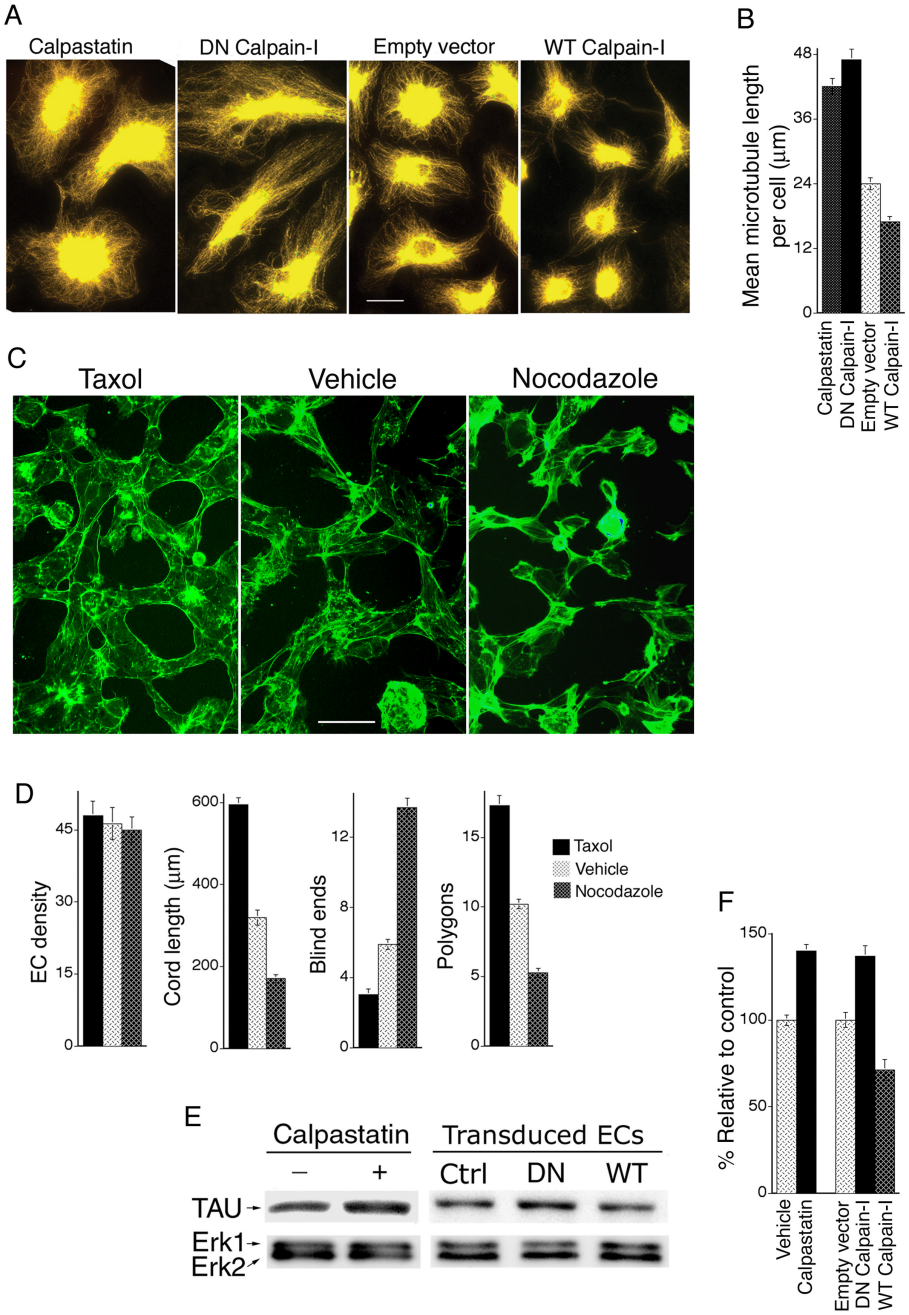
DN calpain-1 supports integration of vascular cord networks by enhancing the microtubule cytoskeleton. (A) MVECs transduced with DN calpain-I or calpastatin peptide (200 nM) exhibited a robust microtubule cytoskeleton relative to Empty Vector controls. In contrast, cells transduced with WT calpain-I exhibited a markedly diminished microtubule cytoskeleton. Microtubules were stained with α-tubulin antibody; bar  = 25 µm. (B) Quantification of mean microtubule lengths (n>20 cells; p<0.001 for DN calpain-I vs. empty vector; p<0.05 for WT calpain-I vs. empty vector; p<0.001 for calpastatin peptide vs. vehicle control). (C) Equal numbers of MVECs were stimulated with collagen-I to undergo capillary morphogenesis (as in Figure 2). Treatment with the microtubule-stabilizing agent taxol (15 µM) during the final hour of cord formation, *i.e.* three hours after the initiation of capillary morphogenesis (see Methods), improved cord formation and reduced blind ends whereas incubation with the microtubule-destabilizing agent nocodazole (15 µM) impaired cord formation and increased blind ends (white arrows). Cords were stained for F-actin with phalloidin; bar  = 50 µm. (D) Quantification of cord organization; n≥17; measured parameters correspond to actual areas of 0.4 mm^2^. Cord length: taxol vs. control p<0.001; nocodazole vs. control p<0.001. Blind ends: taxol vs. control p<0.002; nocodazole vs. control p<0.003. Polygons (closed vascular loops): taxol vs. control p<0.001; nocodazole vs. control p<0.001. (E) As determined with immunoblotting, the microtubule-stabilizing protein tau was increased in MVECs treated with calpastatin peptide or transduced with DN calpain-I, and decreased in MVECs transduced with WT calpain-I relative to empty vector (Ctrl). Total Erk1/Erk2 served as loading controls (see Methods). (F) Quantification of tau protein from blots stained with anti-tau antibody; n≥5. Calpastatin peptide vs. control: p<0.03. DN calpain-I vs. empty vector control: p<0.03. WT calpain-I vs. empty vector control: p<0.05.

Cytoskeletal analyses indicated that MVECs transduced with either DN calpain-I or treated with calpastatin peptide, - a cell permeable and calpain-specific peptide inhibitor representing the active region of the natural calpain inhibitor calpastatin [23], exhibited a robust microtubule cytoskeleton relative to controls (Fig. 3A); quantification of microtubule length indicated a highly significant increase of nearly 100% (Fig. 3B). In contrast, cells transduced with WT calpain-I exhibited a significantly diminished microtubule cytoskeleton (Fig. 3A, B). These observations suggested the possibility that calpain activity regulates integration of MVEC networks through microtubule stability. To test this hypothesis, we performed capillary morphogenesis assays *in vitro* with taxol (15 µM), - a microtubule-stabilizing agent, or nocodazole (15 µM), - an inhibitor of microtubule polymerization. Cords were allowed to form for 3 hours before taxol or nocodazole were added, and cord formation was then allowed to proceed for an additional one hour. Consistent with the importance of microtubule stability for integration of MVEC capillary networks, stabilization of microtubules with taxol markedly improved cord length and reduced blind ends relative to controls (Fig. 3C, D). Conversely, destabilization microtubules with nocodazole reduced cord length and increased blind ends (Fig. 3C, D). Accordingly, taxol-stabilization of microtubules improved vascular network integration *in vitro* similarly to DN calpain-I (Fig. 2), whereas nocodazole-destabilization of microtubules reduced network integration similarly to WT calpain-I (Fig. 2). Therefore, our observations that calpain inhibition enhances microtubules, and that microtubule stability is important for the integration of vascular networks are all consistent with the hypothesis that calpain regulates integration of vascular networks through the microtubule cytoskeleton.

Calpains act through cleavage of numerous intracellular substrates, and many of these substrates regulate the cytoskeleton and cell adhesion [6]. Therefore, we searched for protein targets in MVECs, cultured in the presence of VEGF, that are regulated by DN calpain-I, WT calpain-I, or calpastatin peptide. At the protein level, in >7 separate experiments, we did not detect significant regulation of the focal adhesion proteins paxillin, talin, vinculin, or the cytoskeletal proteins vimentin and α-tubulin, - all of which can be cleaved by calpains [6], [24] (Supporting Information, Fig. S2). However, we consistently observed decreased quantity of the microtubule-stabilizing protein tau in MVECs transduced with WT calpain-I, and increased tau in cells transduced with DN calpain-I and in cells treated with calpastatin peptide (Fig. 3E, F). Tau cleavage by calpain is well documented [6]; and tau is important for microtubule stability [25], [26], Therefore, these data support the hypothesis that inhibition of calpain supports integration of vascular cord networks by increasing tau and, consequently, increasing microtubule stability.

Finally, and consistent with previous findings that calpain suppresses Rho activity by cleaving RhoA to generate a dominant-negative form [27], we found that calpain-inhibitory calpastatin peptide modestly but significantly increased Rho activity in dermal MVECs (Supporting Information, Fig. S3A). Comparably modest increases in RhoA activity have been shown previously to increase actin stress fibers in dermal MVECs [15]. Moreover, consistent with increased Rho activity, calpastatin peptide and DN calpain-I each increased actin stress fibers relative to controls in confluent quiescent cultures of dermal MVECs whereas WT calpain-I nearly abolished stress fibers and (Supporting Information, Fig. S3B). Similarly, in MVECs undergoing cord formation in response to stimulation with collagen I, both calpastatin peptide and DN calpain-I markedly increased actin cable length (Supporting Information Fig. S3C, D). Collectively, these observations raise the possibility that regulation of actin cytoskeletal dynamics, in addition to regulation of microtubule stability, is also important to the mechanism by which calpain regulates capillary morphogenesis and network integration.

### Pharmacological Moderation of VEGF-induced Calpain Activity with Calpain Inhibitor-I Supports Integration of Vascular Networks *In Vitro* and Improves Lumen Formation and Integration of VEGF Neovessels *In Vivo*

**Figure 4:**
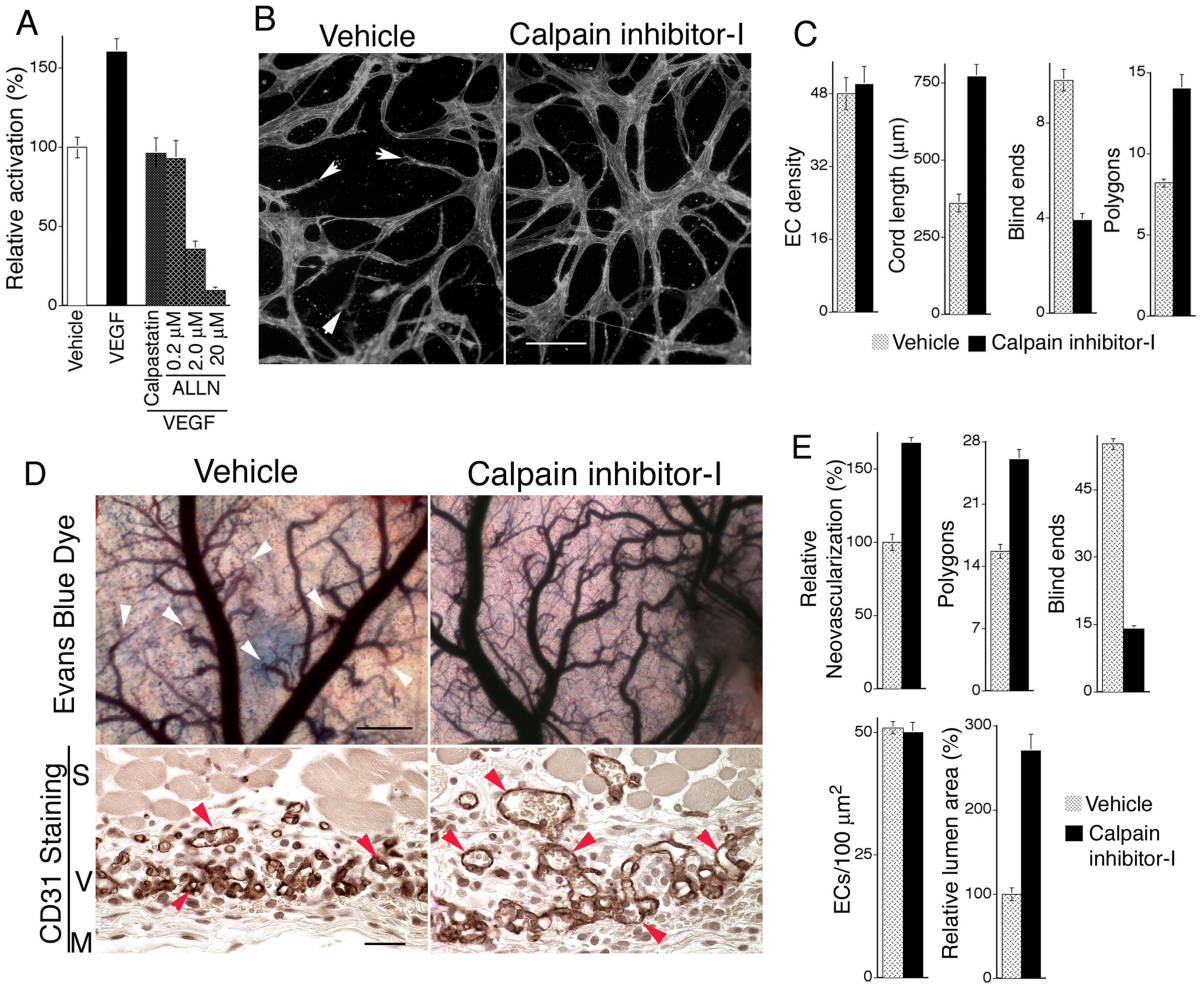
VEGF increases calpain activity in MVECs, and calpain inhibitor-I improves VEGF angiogenesis. (A) MVECs, cultured in the absence of VEGF, were incubated with fluorescent calpain substrate (see Methods) and stimulated with 20 ng/ml VEGF for 15 min; n≥10; control vs. VEGF (p<0.001), VEGF vs. VEGF plus 200 nM calpastatin peptide (p<0.002), VEGF vs. VEGF plus 200 nM ALLN (p<0.002). (B) VEGF-stimulated MVECs undergoing capillary morphogenesis in 3D collagen. Reduction of calpain activity to normal baseline levels with 200 nM calpain inhibitor-I reduced blind ends (white arrows) and markedly improved integration of cord networks. Bar  = 50 µm. (C) Quantification of cord assays shown in (B); n≥15. Measured parameters correspond to values for samples areas of 0.4 mm^2^. Calpain inhibitor-I at the 200 nM dose had no effect on EC density but strongly improved vascular network integration, as indicated by >100% increase in average cord length (p<0.003), >50% reduction in blind ends (p<0.001), and nearly 50% increase in polygons, *i.e.* closed networks (p<0.005). (D) Daily systemic administration of calpain inhibitor-I (10 mg/kg) improves integration and perfusion of new blood vessels. Skin angiogenesis was provoked by VEGF as in Figure 1 but without retroviral packaging cells. Instead animals were treated daily, beginning on day 2, with 10 mg/kg calpain inhibitor-I and harvested on day 8. Evans Blue Dye: images of dermis overlying the Matrigel implants (scale bar  = 250 microns) following perfusion with dye for 10 min, illustrating that calpain inhibitor-I improved blood vessel integration and perfusion (blue color) and reduced blind ends (arrows) relative to vehicle control. CD31 Staining: ECs in cross section stained with CD31 antibody (brown color) illustrating that calpain inhibitor-I improves lumen formation (arrows) relative to control. Scale bar  = 30 microns. S  =  smooth muscle, V  =  region of neovascularization, M  =  Matrigel. (E) Quantification of new blood vessel density, closed vascular networks (polygons), and blind ends from gross images, and EC density and relative lumen area in cross-section from paraffin sections stained with CD31 antibody; n≥17. Gross vessel density (p<0.01), polygons (p<0.03), blind ends (p<0.01), relative lumen area (p<0.01). Numbers of polygons and blind ends correspond to sample areas of 10 mm^2^.

Consistent with previous reports that VEGF induces calpain activity in MVECs from lung and skin [13], [14], we found that VEGF, at the concentration routinely employed in all of our *in vitro* experiments (20 ng/ml), induced calpain activity >50% (Fig. 4A). Calpastatin peptide (200 nM) and another calpain inhibitor, calpain inhibitor-I, also known as ALLN (200 nM), each reduced calpain activity in VEGF-stimulated cells to levels present in the absence of VEGF stimulation. Moreover, 200 nM ALLN, which suppressed calpain activity to baseline identically with 200 nM calpastatin peptide (Fig. 4A), markedly improved integration of vascular cords as measured by reduction in blind ends and increased network connectivity (Fig. 4B, C). However, higher doses of ALLN (≥2.0 µM) did not improve integration of cords, but rather these doses were inhibitory and caused cell rounding (not shown). Notably, these higher doses of ALLN (≥2 µM) severely inhibited calpain activity in comparison with 200 nM ALLN (Fig. 4A), underscoring the importance of moderate calpain inhibition to achieve the desired outcome. To summarize, these experiments indicate that: (1) VEGF induction of calpain activity is likely responsible for impaired integration of vascular cords, and (2) that normalization of calpain activity to baseline levels with calpain inhibitor-I, improves integration of vascular cords comparably to DN calpain-I (Fig. 2). Moreover, these findings suggested that systemic administration of this inhibitor might similarly improve neovascularization *in vivo*. To test this possibility, we employed the same VEGF-driven angiogenesis model described above but without retroviral packaging cells. Instead animals were treated with calpain inhibitor-I (ALLN), which has been used extensively in animal models but for other applications [28], [29], [30], [31]. In initial pilot experiments, calpain inhibitor-I was administered daily (5, 10, 15, and 20 mg/kg, i.p.) beginning on day two following implantation of the VEGF-transfectants. No adverse effects on animal health were observed with any of these doses. As determined grossly in these pilot experiments at day 8, the 20 mg/kg dose clearly inhibited angiogenesis. In contrast, the 10 mg/kg dose did not inhibit neovascularization but rather improved the integration of neovessels. Therefore, more extensive experiments and analyses were performed with the 10 mg/kg dose. As quantified grossly and in cross-section (Figure 4 D, E), 10 mg/kg daily calpain inhibitor-I markedly reduced blind ends and increased vessel lumens similarly to DN calpain-I. These improvements in neovascular architecture were accomplished without any detectable effect on EC density (Figure 4E), indicating that daily administration of 10 mg/kg calpain inhibitor-I did not affect EC number. Similar to DN calpain-I, calpain inhibitor-I (200 nM, ∼1 x IC50) had no effect on production of VEGF by SK-MEL2 cells (the source of VEGF expression) (see Methods), consistent with the fact that VEGF expression was driven constitutively by a CMV promoter. Thus, the cumulative evidence indicates that improvement in neovascular architecture by calpain inhibitor-I is best explained by improvement in capillary morphogenesis rather than by differences in EC density or VEGF expression.

## Discussion

Although defects in neovascular architecture associated with pathological angiogenesis are well recognized (reviewed [2], [3]), mechanistic explanations have been lacking. Experiments described here identify VEGF-induction of calpain activity as an important and previously unrecognized mechanism responsible for failed integration of neovascular networks. As demonstrated here with a mouse skin model of VEGF-driven angiogenesis, expression of an established DN mutant of calpain-I reduced vascular blind ends and markedly improved network integration and vascular perfusion. Conversely, over-expression of WT calpain-I abolished integration of neovascular networks. Importantly, daily administration of calpain inhibitor-I, at 10 mg/kg, markedly improved neovascular network integration and perfusion *in vivo* similarly to DN calpain-I.

Calpains are a complex family, and multiple isoforms may be involved in regulating integration of neovascular networks [6]. The most likely candidates are calpain-I and/or calpain-II which are both are expressed by ECs [32]. Calpain-I is activated by micro-molar calcium whereas calpain-II is activated by milli-molar calcium and by the ERK pathway [6], [33]. VEGF induces calcium uptake and also releases free calcium from intracellular stores [34], [35] suggesting a mechanism for activating calpain-I. VEGF also activates the ERK pathway [36] and activates calpain-II [13], [14]. Our experiments with WT calpain-I establish that over-expression of calpain-I has strong negative consequences for VEGF neovascular network integration both *in vivo* and *in vitro*. Conversely, DN calpain-I and calpain inhibitor-I at the appropriate dose strongly improve network integration. Although both DN calpain-I and calpain inhibitor-I suppressed calpain activity in MVECs as measured with a calpain fluorescent substrate assay, our experiments do not identify which of the various calpain isoforms were inhibited. Nonetheless, and regardless of which calpain isoforms are involved, our studies establish a functional connection between over-exuberant calpain activity and the failed integration of neovascular networks during VEGF-driven angiogenesis.

Importantly, these studies also identify moderate inhibition of calpain activity as a therapeutic strategy to reduce key architectural defects associated with VEGF-driven angiogenesis. Blind ends and poor network integration limit vascular perfusion and seriously reduce the utility of VEGF as a therapeutic agent for tissue revascularization [2]. Abnormal neovessels also contribute to the pathology of disease. For example, in ischemic retinopathies, abnormal neovascularization largely driven by VEGF damages retina and can ultimately lead to blindness [37]. Our findings illustrate that moderate calpain inhibition is a potentially important strategy for rectifying defects in pathological neovessels and also for achieving more functional therapeutic angiogenesis with VEGF. However, it is important to emphasize that appropriate inhibition of calpain is essential for the desired outcome. *In vivo*, we observed inhibition of VEGF-driven angiogenesis with 20 mg/kg daily calpain inhibitor-I, whereas 10 mg/kg daily improved rather than inhibited angiogenesis.

In particular, our *in vitro* experiments established that partial inhibition of calpain activity in MVECs, - specifically by reducing activity to the baseline level present in the absence of VEGF-stimulation, provides marked improvement in vascular cord integration, whereas higher doses of calpain inhibitor impaired cord formation and caused cell rounding. In a previous report, plasmid transfection of aortic ECs with DN calpain-I caused cell rounding [8]; but we did not observe cell rounding in dermal MVECs transduced by retrovirus with DN calpain-I, probably because the level of DN calpain-I expression achieved and consequently the extent of calpain inhibition achieved was more modest. Nonetheless, we did observe cell rounding in dermal MVECs with calpain inhibitor-I, at doses that inhibited calpain activity more severely than retroviral transduction with DN calpain-I. Also, others have reported previously that calpain inhibition can suppress angiogenesis *in vivo* [13] and that calpain inhibition can block capillary morphogenesis *in vitro* [14]. Our findings do not challenge this view; rather they illustrate that more modest calpain inhibition can actually improve network integration. Indeed, suppression of angiogenesis with relatively high doses of calpain inhibitors is not surprising given the importance of calpain activity for basic cellular functions such as adhesion and migration [7], [8], [38], [39]. However, our study illustrates the unanticipated finding that appropriately modest inhibition of calpain activity improves rather than blocks VEGF-driven angiogenesis.

It is also important to emphasize that our experimental findings pertain to VEGF-driven pathological angiogenesis in a mouse model without any disease-related complications. Co-morbid factors such as diabetes and hyper-cholesterolemia cause endothelial dysfunction that seriously hinders neovascularization in response to pro-angiogenic therapy [40], [41]. Thus, in the future, it will be important to determine whether the calpain inhibitor strategy described here also can facilitate VEGF pro-angiogenesis therapy in the presence of endothelial dysfunction due to underlying disease or if such dysfunction must first be addressed with additional, unrelated intervention.

Mechanistically, our *in vitro* experiments indicate that hyper-activation of calpain impairs neovascular network integration and causes blind ends by destabilizing the microtubule cytoskeleton, thereby impairing normal capillary morphogenesis. In particular, our data indicate that reduction of the microtubule-stabilizing protein tau, a well-recognized calpain substrate, is likely responsible for failed neovascular inter-connectivity. Consistent with a critical requirement of microtubules for proper capillary morphogenesis and network integration, we found that microtubule destabilization with nocodazole strongly inhibited integration of cord networks; and these findings are consistent with previous work demonstrating that microtubule de-polymerizing agents induce collapse of new blood vessels *in vivo* and vascular structures *in vitro* [42]. Interestingly, calpain also has been implicated in the collapse of neurite extensions by destabilizing microtubules [43], suggesting a fundamental parallel with findings reported here. Similarly, calpain activation in neurite filipodia slows neurite outgrowth and promotes repulsive growth cone turning [44].

It is important to distinguish microtubule stabilization by tau protein, which is elevated by calpain inhibition, from artificial microtubule stabilization mediated by taxol. Tau-mediated stabilization of microtubules is sensitive to normal regulation by phosphorylation and is therefore dynamic [45], [46]. In contrast, taxol (paclitaxel) hyper-stabilizes microtubules, thereby forming a static tubulin/paclitaxel complex that suppresses the normal dynamic behavior of microtubules [47], [48], [49]. Consistent with reduced microtubule dynamics, taxol has been shown to inhibit angiogenesis [50], [51], [52]; and although we found that taxol improved vascular cord inter-connectivity *in vitro* when administered after cords had begun to form, taxol blocked cord formation if administered prior to the onset of capillary morphogenesis. In contrast, we found that appropriate inhibition of calpain activity improved inter-connectivity of cords even when calpain inhibitors were administered prior to the initiation of capillary morphogenesis.

Other than the microtubule-stabilizing protein tau, we observed no detectable effects of calpain on other known calpain substrates including paxillin, talin, vinculin, vimentin and α-tubulin, - indicating a selectivity for tau in MVECs. However, we did observe a modest but significant reduction in Rho activity, consistent with previous work demonstrating that calpain generates a dominant-negative form of RhoA in ECs [27]. This finding is also consistent with our observations that calpain activity impairs the length and organization of actin stress fibers in MVECs that are regulated by RhoA [15]. In addition, RhoA-mediated actin dynamics are essential for aligning MVECs into capillary cords *in vitro* and also for organizing MVECs into new blood vessels *in vivo* [15]. Thus, calpain-mediated suppression of RhoA activity and actin stress fibers are also likely important to the mechanism by which calpain activity impairs the quality of VEGF-driven angiogenesis. Accordingly, our findings suggest the more general hypothesis that impaired regulation of the EC cytoskeleton is responsible for neovascular defects [3]. Because multiple signaling pathways regulate the cytoskeleton [53], [54], there may prove to be multiple cytoskeletal-targeting strategies, in addition to calpain, for rectifying pathological angiogenesis [3]. Moreover, multiple targeting strategies used in combination may be required to achieve more complete rectification of pathological neovessels.

Finally, because calpain activity regulates neovessel integration during angiogenesis, it seems likely that calpain activity relates to fundamental mechanisms by which sprouting EC tip cells [55] link up with distal sprouting EC tip cells to establish a new contiguous blood vessel. To establish connections with other sprouting ECs at a distance, sprouting tip cells are believed to establish guidance pathways within the extracellular matrix through tensional forces, *i.e.* by creating a “matrical track” [21] or “matrix guidance pathway” [56]. A tensional signal is transmitted through the extracellular matrix by tractional forces exerted by extending tip cells, and the success of this process very likely depends on the dynamic stability of tip cell extensions. Although further study is required to address the involvement of calpain activity directly in this process, it is tempting to speculate that VEGF-activation of calpain activity impairs the stability of tip cell extensions and thereby impairs the ability of these extensions to establish the tensional forces required for linking-up with other sprouting tip cells from distal vessels. This possibility is entirely consistent with the role of calpain activity in tail retraction, *i.e.* in promoting rear end detachment in migrating cells [10] and in promoting neurite retraction [43], as discussed above.

In summary, VEGF activates calpain activity in MVECs resulting in over-exuberant calpain activity that inhibits the integration of neovascular networks, thereby causing formation of vascular blind ends. Moreover, at the cellular level, VEGF-induced calpain activity impairs neovessel integration by destabilizing the microtubule cytoskeleton. Collectively, these experiments identify hyper-activation of calpain as responsible for a previously unexplained neovascular defect commonly associated with pathological angiogenesis, and they provide new understanding as to why VEGF induces abnormal neovascularization. They also identify a previously unrecognized role for calpain activity in regulating blood vessel formation and integration. Finally, these studies identify appropriately modest inhibition of calpain as a previously unrecognized method for improving integration and perfusion of VEGF-induced neovessels. Accordingly, this rational pharmacological strategy offers promise for improving vascular outcomes in clinical settings wherein rectifying defects in pathological neovessels or improving therapeutic angiogenesis is the desired goal [2].

## Materials and Methods

### Reagents

Purified recombinant human VEGF_165_, expressed in Sf21 cells, was obtained from the NCI Preclinical Repository, Biological Resources Branch, Frederick, MD. TAU-5, paxillin, talin, ERK1/2 (K-23), and RhoA antibodies were from Chemicon international (Temecula, CA). Vinculin, vimentin, and α ~-tubulin (Clone DM 1A) antibodies, taxol and nocodazole were from Sigma-Aldrich (St. Louis, MO). ECL Western Blotting Substrate kit was from Pierce (Rockford, IL). Texas-Red secondary antibody for microtubule staining was from Jackson Immunoresearch (West Grove, PA). CD31 (PECAM-1) antibody was from BD Biosciences Pharmingen (San Diego, CA). DAB substrate Kit was from Zymed Laboratory Inc. (Carlsbad, CA). Matrigel and collagen-1 were from BD Biosciences (Bedford, MA). Oregon Green-conjugated phalloidin was from Invitrogen. Calpain inhibitor-I (ALLN) and calpastatin peptide were from Calbiochem (Gibbstown, NJ). Lysine-fixable Texas-red dextran (MW 70 kD) for perfusion studies was from Invitrogen (Carlsbad, CA).

### Preparation of Packaging Cells Expressing Retroviruses Encoding DN Calpain-I and WT Calpain-I

Previously validated DN calpain-I and WT calpain-I cDNAs [8] were subcloned into retrovirus vector pLNCX2 (Clontech, Palo Alto, CA) through HindIII/NotI sites. Clones were verified by sequencing. PT67 retroviral packaging cells (Clontech, Palo Alto, CA), which express the 10A1 viral envelope for production of amphotropic virus, were transfected with pLNCX2 vector containing DN calpain-I, WT calpain-I, or vector without insert (empty vector). Transfectants were cloned, and clones expressing retrovirus at 1×10^5^ c.f.u./ml were selected for subsequent experiments.

### Angiogenesis in Mouse Skin, Retroviral Transduction and Drug Administration *In Vivo*, and Analyses of Vascular Parameters

Neovascularization was investigated *in vivo* according to a previously established method that includes both VEGF_165_-transfected SK-MEL2 cells and retroviral packaging cells, thereby providing a constant source of VEGF and retrovirus [15], [16]. Seven week old female athymic nude mice were injected subcutaneously on right and left flanks with 0.3 ml of 9 mg/ml Matrigel (BD Biosciences) containing 1×10^6^ VEGF_165_-SK-MEL2 cells together with 1×10^6^ retroviral packaging cells, as indicated. Untransfected parental SK-MEL2 cells do not provoke angiogenesis detectably, and therefore the VEGF_165_-SK-MEL2 transfectants employed here allow for specific investigation of VEGF-driven angiogenesis [57]. At times indicated, the animals were euthanized, dissected, and photographed. In addition, to analyze perfusion of new blood vessels, representative animals from each group received tail vein injections of 0.2 ml of 0.5% (w/v) Evan's Blue dye or 70 kD Texas-red dextran (25 mg/ml) in sterile saline. Gross images of perfused vessels were obtained using a Wild M400 Stereomicroscope and SPOT Insight digital camera. For whole mount immunofluorescence analysis of Texas-red dextran tracer-filled vasculature, skin samples were fixed in 4% paraformaldehyde for 4 hr, dehydrated with a graded series of ethanol solutions (50–100%) and cleared with methyl salicylate, mounted in immersion oil, and viewed with a Bio-Rad MRC-1024 Confocal Microscope equipped with an Argon-Krypton Laser. Four-six fields per sample were visualized using x10 objective. For histology, Matrigel implants together with associated skin were fixed for 1 hour in 10% buffered formalin and embedded in paraffin. Immuno-histochemical staining of ECs with CD31 antibody was performed as described [16]. Neovascular density and lumen area were traced through freehand selections on digital images and measured with NIH ImageJ software. EC density was measured through freehand point selections with NIH ImageJ software.

Vascular architecture was also analyzed with Microfil perfusion. The entire vascular tree of the mouse was filled with Microfil MV-122 (Flow Tech; Carver, MA) [58]. The Microfil was allowed to polymerize for 24 h at 4°C. Specimens of flank skin were cleared by dehydration in a graded series of glycerin solutions (50–100%) and photographed using a Wild M400 Stereomicroscope and SPOT Insight digital camera. Blind ends and polygons (closed vascular loops) were quantified through freehand point selections with NIH ImageJ software.

Finally, for experiments with calpain inhibitor-I (ALLN), angiogenesis assays were performed as above but without retroviral packaging cells. ALLN was administered daily i.p. at 10 mg/kg in saline vehicle unless indicated otherwise.

### MVEC Isolation, Cell Culture, and Retroviral Transduction of MVECs

Human dermal MVECs were isolated from neonatal foreskins [59] and cultured [16] in the continuous presence of 20 ng/ml VEGF_165_. All experiments were performed with cells at the fourth to seventh passage. MVECs at passage 5 or less were transduced with retroviruses according to a previously established, efficient method [60]. The transduction procedure was repeated three times on consecutive days before subjecting cells to selection with 300 micrograms/ml G418. This method yields100% transduction as indicated with GFP vectors [15], [16]. Cells were used within one week after selection for experiments.

### Capillary Morphogenesis Assays with MVECs *In Vitro*; Quantification of Parameters

Capillary morphogenesis assays were performed by “overlaying” and “sandwiching” confluent cell monolayers with rat tail collagen-I (BD Biosciences [15], [16]. The sandwich-type assay was performed in 12-well plates with 1.0 mg/ml collagen-I in full medium. Where applicable, calpain inhibitors were added for overnight incubation, prior to adding the upper layer of collagen-I. Capillary morphogenesis was allowed to proceed for 16 h; the assay plates were fixed with 10% formalin for one hour and stained for F-actin with fluorescent Oregon Green-conjugated phalloidin (Invitrogen, final concentration 0.5 units/ml) and subsequently photographed. For the “overlay” assay, calpain inhibitors were added and incubated with cells in 24-well plates overnight. The next day, each well was overlaid with 300 microliters of collagen-I at a concentration of 0.5 mg/ml in serum-free medium together with inhibitors as indicated. Capillary morphogenesis was allowed to proceed for 4 h. For capillary morphogenesis assays involving taxol or nocodazole, cords first were allowed to form for 3 h and then these agents were added, where indicated, for the final hour of the assay. In all cases, cells were fixed in 10% formalin for 10 minutes, permeabilized for one minute with 0.02% Triton-X100 in PBS and stained for F-actin (as above). Cells were photographed with a Nikon inverted fluorescent microscope and digital camera. In all cases, cord length, blind ends, and polygons were quantified using NIH ImageJ software. Cord length was traced and measured through freehand line selections; cords were traced from one edge of the microscopic frame to another, following the least circuitous route. Moreover, cord length was traced and determined independently of intersecting cords. Blind ends and polygons were determined with point selections. Measured parameters correspond to actual areas of 0.4 mm^2^. Statistical analyses were performed as described below.

### Analyses of Cytoskeletal Proteins, Rho Activity, and VEGF Expression

MVECs transduced with DN or WT calpain-I or empty vector were grown to confluence in full medium. Where indicated, calpastatin peptide (200 nM) was added 24 hours before harvest. MVECs were harvested in lysis buffer (20 mM Tris-HCl, 150 mM NaCl, 10% Glycerol, 1% Nonidet P-40, 3 mM MgCl_2_, 1 mM EDTA, 1 mM EGTA, 5 mM Na_3_VO_4_, 150 µM sodium pyrophosphate, plus a cocktail of proteases inhibitors containing 4-(2-aminoethyl)benzenesulfonyl fluoride (AEBSF), pepstatin A, E-64, bestatin, leupeptin, and aprotinin. Lysate (20 µg protein) was subjected to electrophoresis with SDS PAGE on a 4–20% gradient gel. Gels were electrophoretically transferred to PVDF Membrane (BIO-RAD) and stained with antibodies (as indicated). Total Erk1/Erk2 served as loading controls; we have found these proteins to be particularly suitable and preferable as loading controls because they are invariant in MVECs under a variety of experimental conditions [61], [62]. Protein bands were detected with ECL Western blotting substrate. Active GTP-Rho (1×10^7^ ECs/sample) was measured with an established “pull-down” assay followed by immunoblotting [63]. Blots were stained with rabbit polyclonal antibody to RhoA (#SC-179, Santa Cruz Biotechnology, Santa Cruz, CA). Active Rho was quantified with a digital scanner. To assay for any effects of retroviral transduction or calpain inhibitor-I on VEGF expression, VEGF_165_-SK-MEL2 cells were transduced with the various calpain retroviruses (described above) or treated continuously with calpain inhibitor-I (0.2 µM, 2.0 µM). Medium was harvested daily for 8 days, and VEGF_165_ was concentrated with heparin-Sepharose chromatography [64] followed by electrophoresis and immunoblotting with VEGF-specific antibody [65] and quantification with a digital scanner.

### Calpain Fluorometric Assay

Calpain activity was measured in live dermal MVECs with an established fluorescent calpain substrate 7-amino-4-chloromethylcoumarin, *t*-BOC-L-leucyl-L-methionine amide (CMAC, *t*-BOC-Leu-Met; from Invitrogen) [66]; and calpain inhibitors, as indicated, were used to establish specificity. Cells were incubated with 20 µM for 15 min at 37 degrees C; cleavage product was measured with a SpectraMax M5 fluorescent plate reader (excitation/emission 351/430) with SoftMax Pro5 software (Molecular Devices, Sunnyvale CA).

### Statistical Analyses

All data are presented as mean ± S.E.M. Statistical analyses were performed with InStat 3 software for Macintosh, employing the two-tail Mann-Whitney test and assuming unequal variances between the two groups under comparison. In all cases, an individual experimental group was compared with the appropriate control group; and calculated p-values are based on direct comparisons between the two groups, unless indicated otherwise.

## Supporting Information

Figure S1 Angio-architecture of VEGF neovessels as viewed in whole mounts and in cross sections. Tx-Red dextran: Whole mount fluorescent images of the dermal vasculature perfused with TX-Red dextran confirm enhancement in vessel interconnectivity by DN capain-1 and disruption of network integration by WT calpain-I in comparison with Empty Vector control (scale bar  = 100 µm). CD31 Stain: Staining of ECs in cross section with CD31 antibody (brown color) illustrates that DN calpain-I improved lumen formation (red arrowheads) relative to Empty Vector control, whereas WT calpain-I almost completely abolished lumen formation. Scale bar  = 50 µm. See Fig. 1 in the text for higher power views of CD31 staining. S  =  skeletal muscle, V  =  region of neovascularization, M  =  Matrigel.
(9.70 MB TIF)

Figure S2 Survey of potential calpain substrates in dermal MVECs. Unfractionated lysates from equal numbers of MVECs transduced with DN calpain-I, WT calpain-I or empty vector (Ctrl); and control cells treated with calpastatin peptide 24 h prior to harvest were subjected to immuno-blotting and stained with antibodies, as indicated. At the protein level, measurable changes in levels of proteins paxillin, talin, vinculin, or the cytoskeletal proteins vimentin and α-tubulin were not detected. Total Erk1/Erk2 served as loading controls (see Methods).
(2.86 MB TIF)

Figure S3 Calpain regulation of Rho activity and actin stress fibers in dermal MVECs. (A) MVECs treated with calpastatin peptide (200 nM, 24 h prior) exhibited modest but significant increases in Rho activity, consistent with the increase in stress fibers (p<0.05; n = 7). (B) MVECs treated with calpastatin peptide (200 nM, 24 h prior) or transduced with DN calpain-I exhibited increased actin stress fibers relative to Empty Vector controls, as determined with phalloidin staining. In contrast, cells transduced with WT calpain-I exhibited no stress fibers with actin confined to the cell periphery. Bar  = 25 µm. (C) Calpain regulation of the actin cytoskeleton during formation of capillary cords. In all panels, equal numbers of transduced MVECs were stimulated to undergo capillary morphogenesis with collagen-I, and F-actin was stained with phalloidin. Calpastatin peptide and DN calpain-I improved organizational alignment of large actin cables (arrows) and improved formation of capillary cords; in contrast, WT calpain-I disrupted actin organization (arrows) and retarded collagen-induced cord formation. Bar  = 25 µm. (D) Measured lengths of adjoining actin cables in cords; n>19. DN calpain-I vs. control (p<0.001), WT calpain-I vs. control (p<0.003).
(5.92 MB TIF)
