# Supplementary figures and images for: Moderation of Calpain Activity Promotes Neovascular Integration and Lumen Formation during VEGF-Induced Pathological Angiogenesis
Source: PLoS One. 2010 Oct 25;5(10):e13612. doi: 10.1371/journal.pone.0013612 (PMC2963609; doi:10.1371/journal.pone.0013612)

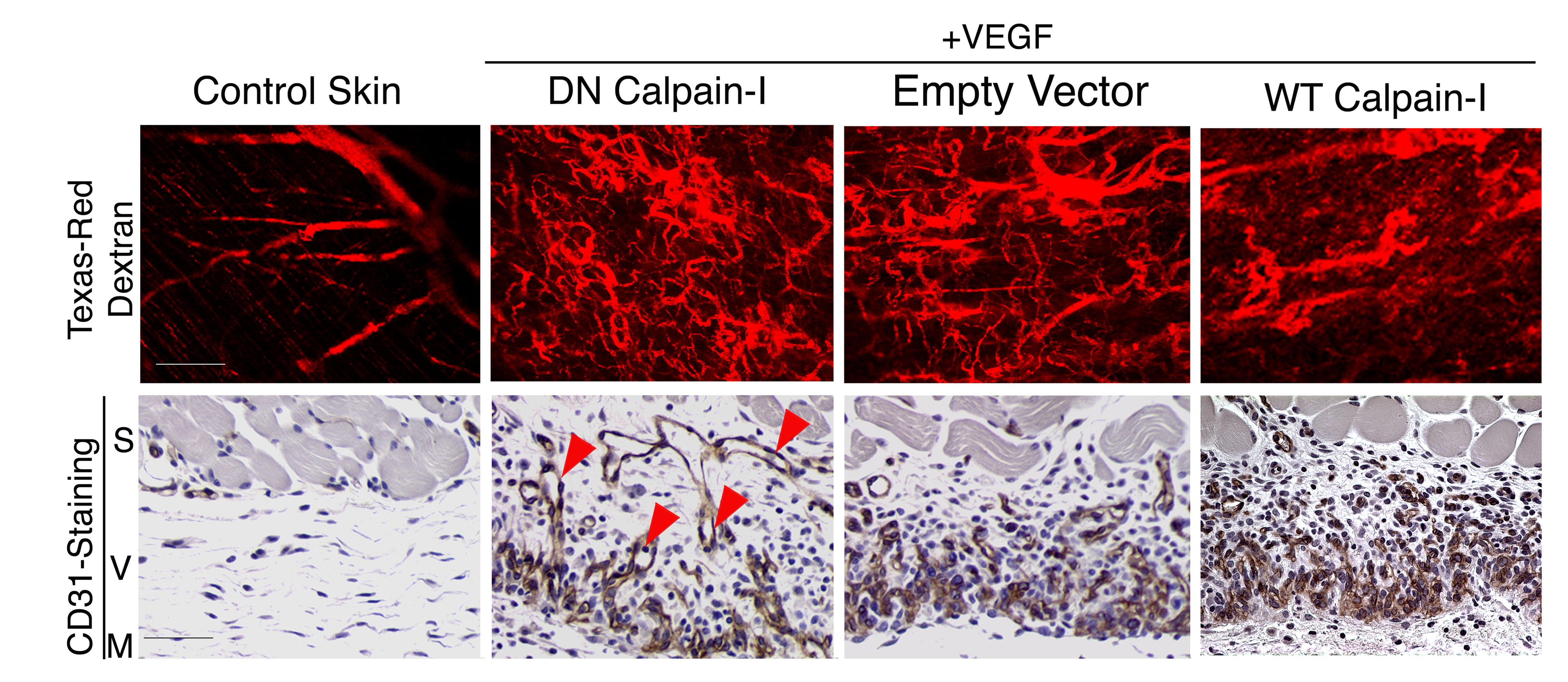

Supplement: Figure S1 — Angio-architecture of VEGF neovessels as viewed in whole mounts and in cross sections. Tx-Red dextran: Whole mount fluorescent images of the dermal vasculature perfused with TX-Red dextran confirm enhancement in vessel interconnectivity by DN capain-1 and disruption of network integration by WT calpain-I in comparison with Empty Vector control (scale bar = 100 µm). CD31 Stain: Staining of ECs in cross section with CD31 antibody (brown color) illustrates that DN calpain-I improved lumen formation (red arrowheads) relative to Empty Vector control, whereas WT calpain-I almost completely abolished lumen formation. Scale bar = 50 µm. See Fig. 1 in the text for higher power views of CD31 staining. S = skeletal muscle, V = region of neovascularization, M = Matrigel. (9.70 MB TIF) [file pone.0013612.s001.tif]

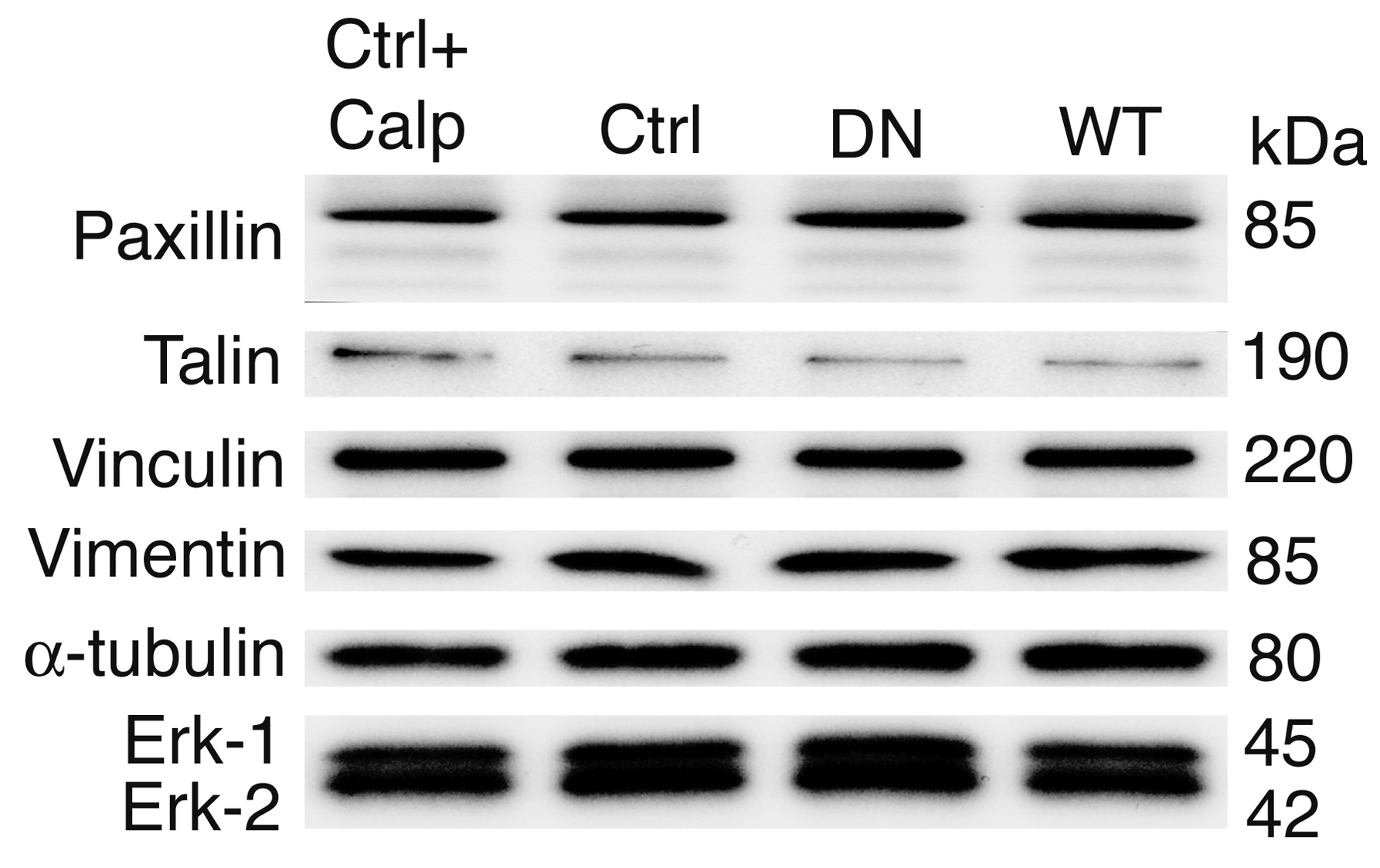

Supplement: Figure S2 — Survey of potential calpain substrates in dermal MVECs. Unfractionated lysates from equal numbers of MVECs transduced with DN calpain-I, WT calpain-I or empty vector (Ctrl); and control cells treated with calpastatin peptide 24 h prior to harvest were subjected to immuno-blotting and stained with antibodies, as indicated. At the protein level, measurable changes in levels of proteins paxillin, talin, vinculin, or the cytoskeletal proteins vimentin and α-tubulin were not detected. Total Erk1/Erk2 served as loading controls (see Methods). (2.86 MB TIF) [file pone.0013612.s002.tif]

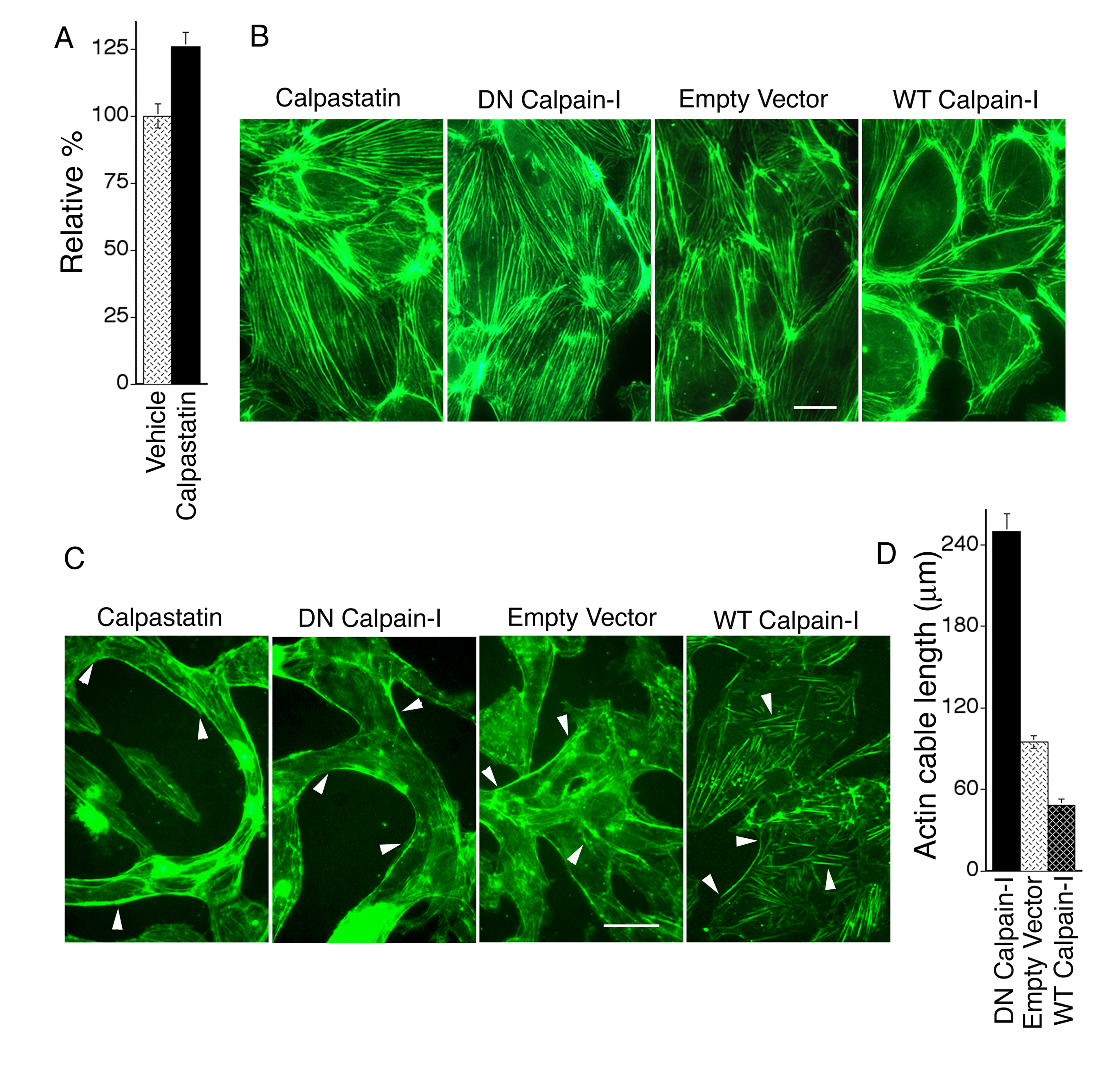

Supplement: Figure S3 — Calpain regulation of Rho activity and actin stress fibers in dermal MVECs. (A) MVECs treated with calpastatin peptide (200 nM, 24 h prior) exhibited modest but significant increases in Rho activity, consistent with the increase in stress fibers (p<0.05; n = 7). (B) MVECs treated with calpastatin peptide (200 nM, 24 h prior) or transduced with DN calpain-I exhibited increased actin stress fibers relative to Empty Vector controls, as determined with phalloidin staining. In contrast, cells transduced with WT calpain-I exhibited no stress fibers with actin confined to the cell periphery. Bar = 25 µm. (C) Calpain regulation of the actin cytoskeleton during formation of capillary cords. In all panels, equal numbers of transduced MVECs were stimulated to undergo capillary morphogenesis with collagen-I, and F-actin was stained with phalloidin. Calpastatin peptide and DN calpain-I improved organizational alignment of large actin cables (arrows) and improved formation of capillary cords; in contrast, WT calpain-I disrupted actin organization (arrows) and retarded collagen-induced cord formation. Bar = 25 µm. (D) Measured lengths of adjoining actin cables in cords; n>19. DN calpain-I vs. control (p<0.001), WT calpain-I vs. control (p<0.003). (5.92 MB TIF) [file pone.0013612.s003.tif]
